# The effects of reproductive specialization on energy costs and fitness genetic variances in cyclical and obligate parthenogenetic aphids

**DOI:** 10.1002/ece3.247

**Published:** 2012-07

**Authors:** Mauricio J Carter, Jean-Christophe Simon, Roberto F Nespolo

**Affiliations:** 1 Department of Animal and Plant Sciences, University of SheffieldWestern Bank, Sheffield, United Kingdom; 2 INRA, UMR 1349 Institut de Génétique, Environnement et Protection des Plantes (IGEPP) Domaine de la Motte – 35653 Le Rheu CedexFrance; 3 Instituto de Ciencias de la Tierra y Evolución, Facultad de Ciencias, Universidad Austral de ChileChile

**Keywords:** Cyclical parthenogenesis, G-matrix, quantitative genetics, sexuality, standard metabolic rate

## Abstract

Organisms with coexisting sexual and asexual populations are ideal models for studying the consequences of either reproductive mode on the quantitative genetic architecture of life-history traits. In the aphid *Rhopalosiphum padi*, lineages differing in their sex investment coexist but all share a common parthenogenetic phase. Here, we studied multiple genotypes of *R. padi* specialized either for sexual and asexual reproduction and compared their genetic variation in fitness during the parthenogenetic phase. Specifically, we estimated maintenance costs as standard metabolic rate (SMR), together with fitness (measured as the intrinsic rate of increase and the net reproductive rate). We found that genetic variation (in terms of broad-sense heritability) in fitness was higher in asexual genotypes compared with sexual genotypes. Also, we found that asexual genotypes exhibited several positive genetic correlations indicating that body mass, whole-animal SMR, and apterous individuals production are contributing to fitness. Hence, it appears that in asexual genotypes, energy is fully allocated to maximize the production of parthenogenetic individuals, the simplest possible form of aphid repertoire of life-histories strategies.

## Introduction

One of the most important attempts to formalize a general theory in biology was Fisher's fundamental theorem of natural selection (FTNS), according to which the instantaneous rate of increase in the mean fitness of a population equals its additive genetic variance in fitness (Fisher 1930; Okasha 2008; Bijma 2010). However, the realism of the theorem has been questioned as the FTNS predicts a continuous increase in fitness maintaining all other things equal (including the environment and the average effects of alleles) (Bijma 2010). Among other things, nonadditive genetic variation and resource limiting leading to competition and deterioration of the environment are common factors in natural populations that violate these assumptions. Then, the average fitness of the population cannot always increase (Okasha 2008). The initial and perhaps most popular empirical support for the Fisher fundamental theorem was based on the fact that life histories have low heritability because they are constant targets of directional selection (Gustafsson 1986; Roff and Mousseau 1986; Mousseau and Roff 1987). However, this was later debated because of the neglected contribution of environmental variation that life-history traits accumulate (Price and Schluter 1991; Merila and Sheldon 2000; Kruuk et al. 2003).

The modern interpretation of the FTNS, which considers the partial change in mean fitness under a constant environment (Okasha 2008), has renewed the interest in the quantitative genetics of fitness traits (Reale and Festa-Bianchet 2000; Crow 2002; Bolnick and Nosil 2007; Okasha 2008; Ewens 2011; Guillaume 2011; Hendry et al. 2011). The FTNS can thus be used as a null hypothesis for the empirical study of adaptation, permitting to test its assumptions in experimental settings (Reale and Festa-Bianchet 2000). With this interpretation, empirical supports for the FTNS became varied (Kruuk et al. 2000; Reale and Festa-Bianchet 2000; McCleery et al. 2004; Bolnick and Nosil 2007).

Although several authors already advocated the use of cyclic parthenogenetic organisms for the study of the fitness consequences of sexual reproduction (Colegrave et al. 2002), few experimental studies have addressed in these organisms the problem of genetic variation in fitness, experimentally (Pfrender and Lynch 2000; Morgan et al. 2001). In cyclically reproducing organisms, sexuality is a phenotypically plastic trait that can be triggered by environmental conditions (De Meester et al. 2004). This is the case of aphids, for which the alternance of parthenogenetic (asexual) and sexual reproduction has important consequences on the genetic structure and architecture of natural populations (Simon et al. 1991; Simon et al. 2002; Halkett et al. 2008; Nespolo et al. 2008; Nespolo et al. 2009). In fact, parthenogenesis is the main reproductive mode of aphids while sexual reproduction is triggered once a year to anticipate stressful conditions of cold winter. During warm periods aphids reproduce asexually (producing viviparous clonal offspring) exhibiting high population growth rates (Rispe et al. 1996). When the cold season begins, sexual morphs are produced, which mate and lay frost-resistant eggs (Rispe et al. 1996). Thus, the main known advantage of sexual reproduction is the resistance to freezing conditions, which typically kill the asexual forms (Newton and Dixon 1988; Simon et al. 1991; Rispe et al. 1996; Simon et al. 2002; Nespolo et al. 2009).

In its distribution range, the bird cherry-oat aphid, *Rhopalosiphum padi* (L.) displays genotype specialization associated to reproductive mode variation (Simon et al. 1991; Hulle et al. 1999), which has consequences on its life-history patterns, dispersal traits, and reproductive investment (Simon et al. 1991; Simon et al. 2002). Two kinds of genotypes can be identified in terms of their capacity for producing sexual morphs: “asexual” (i.e., sustained asexuality without a full commitment to the sexual reproduction) and “sexual” (i.e., obligate and regular alternation of asexual and sexual reproduction) genotypes. Sexual and asexual genotypes share a common summer parthenogenetic phase on cereals and other grasses. However, they live on separate hosts the remaining time because sexual reproduction is associated with host shift in this aphid. Earlier studies showed that asexual lineages may originate from sexual ones through diverse mechanisms so that they form a mixed array of phylogenetically distinct genotypes (Delmotte et al. 2003). Hence, *R. padi* constitutes a good system to study the impact of reproductive mode variation on the genetic architecture of life-history traits because it allows within and between comparisons of sexual and asexual lineages with distinct genetic backgrounds (Delmotte et al. 2003; Nespolo et al. 2008). While the population genetics and phylogeny of sexual and asexual genotypes of *R. padi* have been addressed in several studies, little attention has been paid to their respective ecology and fitness. In particular only few works have studied the life histories of reproductive specialization in *R. padi*. In previous works, Nespolo and co-workers determined the effects of temperature on asexual and sexual morph production (Nespolo et al. 2008) and the life-history consequences of phase induction (Nespolo et al. 2009). Both studies were performed on the basis of genetic variances in several traits (i.e., comparing “G matrices”), and showed that sexuality and reproductive mode variation have profound effects on the structure of the G matrices. However, both studies analyzed an undifferentiated array of sexually and asexually specialized genotypes. Given the strong and consistent pattern of reproductive specialization that *R. padi* exhibits (Halkett et al. 2006), it is expected that during the shared parthenogenetic phase, asexually specialized genotypes would be benefited compared with sexually specialized ones, since the latters are benefited in winter (Moran 1992; Rispe et al. 1996). In fact, previous studies have indicated that asexual genotypes maximize their survival and fecundity under these conditions, which can be interpreted as being “adapted” to such (summer) conditions (Rispe et al. 1996; Hulle et al. 1999). The FTNS tells “the rate of increase in fitness of any organism at any time is equal to its genetic variance in fitness at that time” (Edwards 1994). Hence, if asexual genotypes are in the process of being “adapted” to summer conditions, its average fitness should be increasing (i.e., genetic variance or heritability of fitness should be higher than zero). On the other hand, if asexual genotypes are already adapted, being fixed all their alleles, then average population fitness should not longer increase (i.e., genetic variance or heritability of fitness should not different than zero). Seasonality and population subdivision would promote the former conditions, as populations would never attain the required equilibrium conditions for the adaptation to be fixed. During summer, sexual genotypes would be at disadvantage compared with asexual genotypes, and hence the average increase of population fitness would be very low or undetectable (i.e., genetic variance or heritability of fitness should not different than zero). Also, several specific features of the adaptation process would appear in the asexual genotypes, compared with the sexual ones. For instance, the allocation of energy to less costly asexual phenotypes could be translated into lower energy costs. However, it is not clear what to expect during the asexual phase, where sexual genotypes are not constrained to produce sexual forms. One rationale would be that the genetic program of sexual genotypes maintains its constraints even when not producing sexual forms, thus reproducing at a higher energy cost during the parthenogenetic phase, compared with asexual genotypes. Hence, higher energy metabolism would be expected in sexual genotypes.

It is important to note that differential patterns of energy allocation, could be reflected either in direct energy measurements (i.e., metabolic rate) or in differences of body mass (i.e., somatic tissue). In order to test these predictions, we compared asexual and sexual genotypes during the asexual phase, in *R. padi*.

## Material and Methods

### Biological system

**Figure 1:**
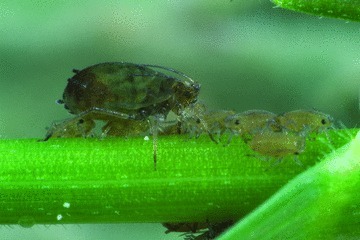
Model organism *Rhopalosiphum padi* during parthenogenetic (viviparous) reproduction. Photo by Bernard Chaubet.

Our biological model was the bird cherry-oat aphid (*R. padi*; Fig. 1). As typical for aphids, “sexual” genotypes of *R. padi* reproduce through cyclical parthenogenesis, whereby a parthenogenetic phase on many species of Poaceae (cereals and other grasses) alternates with a sexual phase on a specific winter host, which is the tree species *Prunus padus L.* (Simon et al. 1991; Rispe et al. 1999). The shift from parthenogenetic to sexual reproduction occurs by the end of summer and is triggered by increasing night length and decreasing temperature (Dixon and Glen 1971). By contrast, asexual genotypes reproduce all year long parthenogenetically on a wide range of Poaceae plants. Sexual and asexual genotypes generally coexist in regions where cold and warm winters fluctuate, which is the case of western France (Simon et al. 1991; Halkett et al. 2008).

**Figure A1:**
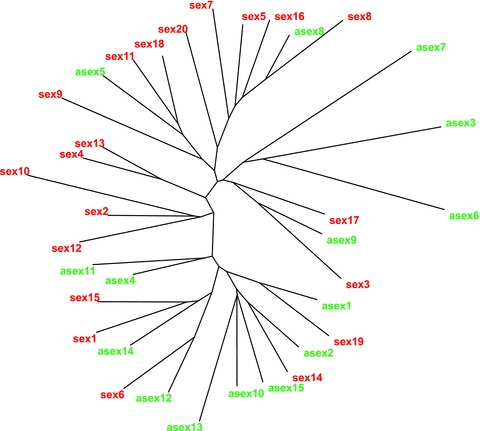
Neighbour-joining tree based on the allele shared distance (DAS, Chakraborty and Jin 1993) matrix calculated with eight microsatellite loci for the 35 sexual and asexual multilocus genotypes of *Rhopalosiphum padi* used for fitness and energetic measurements.

From end of fall to late spring, asexual and sexual genotypes are clearly separated in space, being on Poaceae and on *P. padus*, respectively. This spatial separation conveniently allows ascertaining sexual and asexual genotypes since no morphological characters exist to distinguish between them. Asexual genotypes were sampled by end of fall beginning of winter on barley or wheat plants separated by at least 1 m in order to minimize collection of aphids from the same clonal genotype. Sexual genotypes were collected on *P. padus* after they had just hatched from eggs, ensuring that they did not undergo asexual reproduction. To initiate clonal (isofemale) genotypes in the laboratory, parthenogenetic females of asexual and sexual genotypes of *R. padi* were collected respectively in Pleine-Fougères (Brittany, France) in the vicinity of Mont-Saint Michel in autumn 2006 and 2007, and in La Chapelle Thouarault (Brittany, France) in the vicinity of Rennes in spring 2008. Each parthenogenetic female was kept isolated on a wheat plant and allowed to establish an isofemale genotype. Asexual and sexual genotypes reared in the laboratory were then genotyped at eight microsatellite markers to characterize their multilocus genotype or MLG (see Halkett et al. 2008 for methodological aspects). Fifteen asexual genotypes and 20 sexual genotypes were then selected for the experiments based on their MLG (Table A1). Because clonal copies of the same genotype are frequent in asexual lineages of *R. padi* (Delmotte et al. 2002), we were constrained in the number of asexual genotypes. Genetic analysis also permitted to check that aphid genotypes used here did not belong to differentiated genetic clusters associated with either reproductive modes but rather represented an array of sexual and asexual lineages with distinct genetic backgrounds (Fig. A1). Each genotype was reared in isolation under conditions ensuring continuous parthenogenetic reproduction, during ca. 20 generations (16 h light:8 h dark, 18°C) prior experiments. Wheat was used for both rearing and experiments.

### Fitness measurements

Sexual and asexual genotypes were monitored in controlled conditions to assess various life-history traits. Experimental conditions for fitness measurements were set at 18°C and 16 h of light to ensure sustained parthenogenetic reproduction in both sexual and asexual genotypes of *R. padi* (Dixon and Glen 1971). To isolate maternal and grand-maternal effects, we started rearing parthenogenetic females of each genotype in isolation, two generations before the experiments. For trait measurements, we kept the clonal genotypes at the rearing conditions and we assessed, on eight replicates per genotype, the following traits: age at maturity of focal individuals (number of days elapsed from their birth to the first offspring produced) and fecundity of winged ( = production of *winged* individuals from the focal individual) and wingless ( = production of *wingless* individuals from the focal individual). With this information, we constructed life tables and estimated fitness as the intrinsic growth rate (***r***), by means of the discrete version of the Euler–Lotka equation, 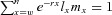, and by computing *lifetime reproduction* of each focal individual, as the total number of individuals produced during its lifetime.

### Standard metabolic rate (SMR)

As indicated for animals with very low rate of metabolism, rates of carbon dioxide production (VCO_2_) were used as measure of SMR (Lighton 2008). VCO_2_ was determined using “closed system” metabolic chambers (Lighton 2008), consisting of glass 2.5 mL hermetic syringes fitted with three-way valves (see also Chappell 1983; Ashby 1997). Animals were weighed (adult body mass = *M*_b_) to the nearest micrograms with a microbalance and then placed, individually, in the syringes. The syringes were sealed from the atmosphere and placed in a temperature controlled, dark incubator for the duration of the measurement period (ca. 90 min). Three blank syringes served as controls for each series of measurements. We injected the air of the syringe into a tube connected to the CO_2_ analyzer after passing through CO_2_-absorbent granules of Baralyme™ and Drierite™. At the end of the measurement interval, CO_2_ concentrations were determined using an infrared CO_2_ analyzer Li-Cor 6251 (Lincoln, NV, USA) capable of resolving difference of 1 part per million (ppm) of CO_2_ in air. We computed the total amount of CO_2_ produced per aphid as the area below the curve of CO_2_ concentration in time. Then, the measures were transformed from ppm to mL CO_2_ h^−1^, taking into account the flow rate and incubation time for each syringe. Therefore, each measurement is an average of CO_2_ production over several hours. According to previous measurements, technical errors associated with this measurement method are small (Castaneda et al. 2010a,b), and its simplicity allows simultaneous measurements of a large number of individuals. In this experiment, we measured SMR for a total of ca. 312 individual aphids.

### Statistics I: quantitative genetic parameters

Common statistical analyses were performed using Statistica 6.0 software (2006). Our experimental design based on clonal genotypes, with the trait of interest assayed in the progeny, allowed us to estimate two variance components: **G**, the (broad sense) genetic variance, and **E**, the specific environmental variance or residual variance. We used a restricted maximum-likelihood based method, the animal model procedure (Boldman et al. 1995) implemented in the MTDFREML software. Iterations were continued until the differences in successive likelihood were less than 0.00001. We iterated the analysis in the full model (**GE**) and several starting values were assayed to ensure the solution being actually a global maximum of the likelihood. Also, we tested the pure environmental model by constraining the **G** parameter of the full model to zero, which gives a new likelihood value. The statistical significance of the **G** component was assessed using likelihood-ratio tests (LRTs), where critical χ^2^ is selected with degrees of freedom equal to the number of parameters dropped from the model (i.e., df = 1; χ^2^*crit* = 3.841). Genetic variances and covariances were estimated in a similar fashion, but including pairs of traits in a multivariate version of the animal model software (Boldman et al. 1995). We also present asymptotic standard errors (i.e., estimated values for large samples).

### Statistics II: G-matrices comparison

The experimental treatment was sexual/asexual genotypes. The resulting **G** matrices were first compared using the Flury hierarchical method, using the software CPCrand (Phillips and Arnold 1999). This procedure is a principal component approach to the comparison of matrices (Flury 1988), whose application to **G** matrices was developed by Phillips and Arnold (1999). The method, based on maximum likelihood, compares two or more matrices and determines their structural differences by comparison of eigenvectors (orientation of axes of the principal component analysis) and eigenvalues (variance along each axis) in a hierarchical fashion. This progression tests (following this order): (1) unrelated structure, indicating that matrices do not share any eigenvector, (2) partial common principal component, indicating that matrices share some eigenvectors, (3) common principal components, indicating that matrices share all eigenvectors, but not eigenvalues, (4) proportionality, indicating that matrices share all eigenvectors and eigenvalues all differing by a scalar amount, and (5) equality, indicating that matrices share all eigenvector and eigenvalues (see also Steppan et al. 2002). An LRT was calculated for each model against the model of “unrelated structure” (the “jump-up” approach; see Phillips and Arnold 1999). A randomization was performed to test the null hypothesis that the model fits the data significantly better than the unrelated structure. We performed 5000 iterations, each randomly assigning genotypes to the compared group. We also compared the **G** matrices by the Jackknife–MANOVA method, which was performed by a script provided by D. Roff, and run in the S-plus software (version 2000). This procedure, developed by Roff (Roff 2002a, 2006), uses the Jackknife procedure to produce a distribution of pseudovalues of matrix elements within each group. These pseudovalues are produced by deleting each sampling unit (genotypes, in this case) in turn. The final data matrix is arranged such that the columns comprise the pseudovalues of each covariance and the rows are the results of the deletion of a given genotype (Roff 2006).

## Results

### Trait means

**Figure 2:**
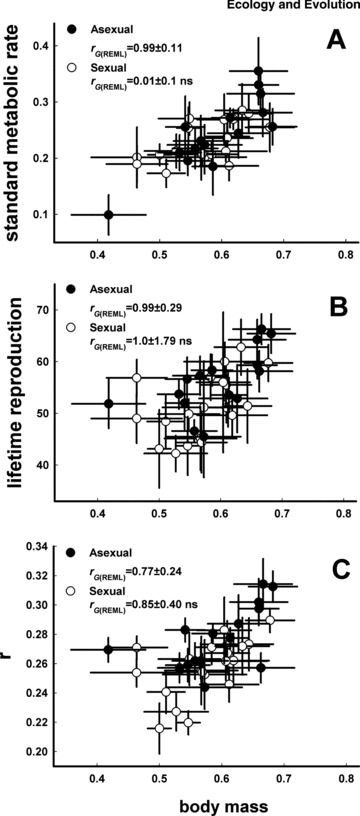
Clonal means in sexual and asexual genotypes of *R. padi* for: body mass and (A) standard metabolic rate (SMR), (B) total fecundity, (C) fitness (per-capita intrinsic growth rate). The genetic correlations for sexual and asexual genotypes are presented as a reference (see detailed statistics including the genetic correlation for the whole population in Table 3).

**Figure 3:**
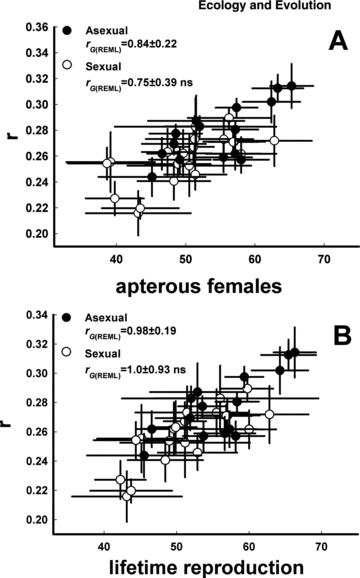
Clonal means in sexual and asexual genotypes of *R. padi* for: (A) apterous female production and fitness; and (B) total fecundity and fitness. The genetic correlations for sexual and asexual genotypes are presented as a reference (see detailed statistics including the genetic correlation for the whole population in Table 3).

Pairwise comparisons between sexual and asexual genotypes showed that production of apterous females, lifetime reproduction, and the intrinsic rate of increase (***r***) were significantly higher in asexual compared with sexual genotypes (i.e., reproductive specialization; see Table 1). Both types of reproductive variants showed important inter- and intraclone variation in all measured traits (Figs. 2 and 3).

**Table 1:** Phenotypic means (±SE from genotype means) of traits measured in asexual and sexual genotypes of *R. padi* in conditions of sustained parthenogenetic reproduction (see Methods for details). Nested-ANOVA was performed on clonal lines nested into each kind of reproductive mode. Significant effects were highlighted in bold and indicated by: *P < 0.05; **P < 0.01; ***P < 0.001

Trait	Asexual genotype Mean (±SE)	Sexual genotype Mean (±SE)	Clonal line *F*_(33,242)_	Sexual/asexual *F*_(1,242)_
Standard metabolic rate, SMR (mL CO_2_ h^−1^)	0.25 ± 0.009	0.22 ± 0.007	2.044**	1.925
Adult body mass, *M*_b_ (mg)	0.59 ± 0.011	0.57 ± 0.010	1.947**	1.531
Age at maturity, AM (days)	8.33 ± 0.078	8.45 ± 0.074	1.947**	0.350
Apterous females, AF (individuals)	54.0 ± 1.237	49.7 ± 1.269	1.555*	6.305*
Winged females, VL (individuals)	1.95 ± 0.434	1.47 ± 0.390	1.321	0.716
Lifetime reproduction, LR (individuals)	55.9 ± 1.170	51.2 ± 1.232	1.403	7.190**
Intrinsic rate of increase, *r*	0.27 ± 0.003	0.25 ± 0.003	2.236***	18.22***

### Heritabilities and genetic correlations

In general, we found low levels of genetic variances (heritabilities being less than 0.20) in both reproductive variants, being somewhat higher in the asexual genotypes, where only SMR and the intrinsic rate of increase (***r***) resulted significant (Table 2). No significant heritability was found in sexuals and only three were found in asexuals: SMR, body size, and ***r***. The fact that a genetic correlation existed between SMR and body size (see below) suggests that the heritability in SMR is a consequence of such correlation. Indeed, computing heritability using body mass as a covariable yields low and nonsignificant heritability (data not shown). In the pooled sample, age at maturity also showed significant heritability (Table 2).

**Table 2:** Broad-sense heritabilities of a suite of life-history traits computed on sexual and asexual genotypes of *R. padi* and measured in conditions of sustained parthenogenetic reproduction (see Methods for details). Significant values after REML and likelihood-ratio test with one degree of freedom are indicated with asterisks (χ^2^ values are presented in parenthesis with: *P < 0.05, **P < 0.01, and ***P < 0.001) and highlighted in bold

Trait	Asexual genotypes (*N* = 15) REML (±SE)	Sexual genotypes (*N* = 20) REML (±SE)	Pooled genotypes (*N* = 35) REML (±SE)
Standard metabolic rate (SMR)	0.18 ± 0.08 (10)**	0.001 ± 0.05 (0.01)	0.11 ± 0.05(7.83)**
Adult body mass (*M*_b_)	0.14 ± 0.08 (5.29)*	0.06 ± 0.06 (0.23)	0.10 ± 0.05(7.12)**
Age at maturity (AM)	0.08 ± 0.06 (1.74)	0.11 ± 0.073 (0.09)	0.10 ± 0.05(6.23)*
Apterous females (AF)	0.06 ± 0.06 (3.33)	0.04 ± 0.05 (0.07)	0.07 ± 0.04 (3.44)
Winged females (VL)	0.03 ± 0.04 (0.90)	0.04 ± 0.05 (0.89)	0.04 ± 0.04 (1.07)
Lifetime reproduction (LR)	0.06 ± 0.06 (1.31)	0.01 ± 0.05 (1.12)	0.07 ± 0.04 (3.62)
Intrinsic rate of increase (*r*)	0.19 ± 0.09 (3.91)*	0.08 ± 0.06 (0.87)	0.17 ± 0.05(16.21)***

The genetic correlations that resulted significant either for the whole sample or sexual and/or asexual genotypes are graphed in Figs. 2 and 3 (see also Table 3). We found seven large and positive genetic correlations in the asexual genotypes, which involved SMR, *M*_b_, lifetime reproduction, apterous individual production, and ***r*** (Table 3). This contrasted with the outcome of sexual genotypes, where large intraclone variation produced only nonsignificant genetic correlations (Table 3; Figs. 2 and 3). Among them, the large positive genetic correlation between *M*_b_ and both fitness measures in asexuals (Fig. 2) suggests a strong association between body size and reproductive output in asexual genotypes. Also, both fitness measures showed a positive genetic correlation in asexual genotypes (Fig. 3) (Table 3).

**Table 3:** Genetic correlations computed with REML on a suite of traits measured in sexual and asexual genotypes of *R. padi* in conditions of sustained parthenogenetic reproduction (see Methods for details). Significant values after REML and likelihood-ratio test with one degree of freedom are indicated with asterisks (χ^2^ values are presented in parenthesis with: *P < 0.05, **P < 0.01, and ***P < 0.001) and highlighted in bold. Abbreviations are as in Tables 1 and 2

Trait	Asexual genotypes (±SE)	Sexual genotypes REML (±SE)	Pooled genotypes REML (±SE)
SMR–*M*_b_	0.99 ± 0.11 (12.47)**	0.01 ± 0.1 (0.23)	0.99 ± 0.11 (12.61)***
SMR–AM	−0.39 ± 0.47 (0.59)	0.12 ± 0.1 (1.46)	−0.43 ± 0.32 (1.49)
SMR–APT	0.19 ± 0.09 (3.11)	0.02 ± 0.2 (0.08)	0.87 ± 0.35 (4.74)*
SMR–VL	−0.05 ± 0.68 (0.08)	0.04 ± 0.1 (0.57)	0.07 ± 0.51 (0.02)
SMR–LR	0.94 ± 0.37 (3.86)*	0.001 ± 0.1 (0.10)	0.91 ± 0.35 (5.25)*
SMR–*r*	0.66 ± 0.28 (3.91)*	0.05 ± 0.6 (0.14)	0.70 ± 0.21(5.79)*
*M*_b_–AM	−0.10 ± 0.51 (0.03)	−0.55 ± 0.48 (0.01)	−0.42 ± 0.32 (1.39)
*M*_b_–APT	0.85 ± 0.27 (3.12)	0.86 ± 0.68 (0.03)	0.98 ± 0.21 (6.93)*
*M*_b_–VL	0.07 ± 0.77 (0.11)	0.66 ± 0.84 (0.35)	0.66 ± 0.59 (1.55)
*M*_b_–LR	0.99 ± 0.29 (4.01)*	1.0 ± 1.79 (0.11)	1.0 ± 0.31 (0.03)
*M*_b_–*r*	0.77 ± 0.24 (3.87)*	0.85 ± 0.40 (0.05)	0.81 ± 0.17 (8.10)**
AM–APT	−0.10 ± 0.64 (0.23)	−0.81 ± 0.66 (1.98)	−0.60 ± 0.39 (2.01)
AM–VL	−0.81 ± 0.65 (1.47)	−0.61 ± 0.64 (0.83)	−0.97 ± 0.53 (4.35)*
AM–LR	0.40 ± 0.60 (0.37)	−1.0 ± 1.26 (0.03)	−0.88 ± 0.36 (4.66)*
AM–*r*	−0.70 ± 0.29 (2.48)	−0.44 ± 0.44 (0.19)	−0.57 ± 0.22 (3.80)*
APT–VL	−0.42 ± 0.80 (0.23)	1.0 ± 0.62 (2.11)	0.01 ± 0.62 (0.18)
APT–LR	0.97 ± 0.05 (1.63)	1.0 ± 0.35 (1.41)	0.01 ± 1.56 (0.22)
APT–*r*	0.84 ± 0.22 (3.92)*	0.75 ± 0.39 (0.89)	0.93 ± 0.16 (8.07)**
VL–LR	−0.20 ± 0.95 (0.04)	−1.0 ± 1.17 (0.01)	0.001 ± 0.60 (0.01)
VL–*r*	0.25 ± 0.67 (0.14)	0.60 ± 0.80 (0.66)	0.66 ± 0.49 (2.21)
LR–*r*	0.98 ± 0.19 (4.56)*	1.0 ± 0.93 (0.78)	1.0 ± 0.99 (0.99)

### Matrix comparisons

The differences between G matrices were not large, as we did not find any change in the sign of genetic covariances. Whereas the Flury method of P-matrix comparison yielded nonsignificant differences, the G-matrix comparison found that matrices shared their principal component structure (Table 4). However, the Jackniffe–MANOVA method indicated that matrices differed significantly (Table 4). Patterns of differentiation of asexual genotypes were explained by their larger values of genetic variance in SMR, fitness measures and its genetic correlations with *M*_b_, total fecundity, and apterous parthenogenetic females, compared with sexual genotypes. However, the P-matrix comparison did not show significant differences (Table 4).

**Table 4:** G–P matrices comparisons between asexual and sexual lineages of *R. padi* using the Flury hierarchy and the MANOVA methods. The verdict of the Flury hierarchy is the model that best explains the difference between two matrices. The probabilities given for the Flury hierarchy correspond to the test of equality of two matrices, not necessarily to the verdict. The method MANOVA refers to the results obtained by using the scores of the pseudovalues of the G and P matrices. The numbers in parentheses for the MANOVA method correspond to the freedom degree associated to respective test

	Flury Hierarchy			MANOVA Jackknife		
		
	LRT	*P*-value	Verdict	λ (Wills)	*F*-value	*P*-value
**G matrix**						
Asexual–sexual	3.116	<0.05	PCPC 2	0.045	17.06_(21,17)_	<0.001
**P matrix**						
Asexual–sexual	5.647	0.80	Equal	0.905	1.27_(21,255)_	0.19

## Discussion

A general indicator of how a population is adapting to the environment (assuming that the environment does not change) derives from the FTNS: the rate of increase in mean fitness in a population equals its genetic variance in fitness (Fisher 1930; Gustafsson 1986; Frank and Slatkin 1992). In other words, the genetic variance in fitness of a given population would represent an instantaneous indicator of its mean rate of increase. In our experiment, we compared the quantitative genetics of life-history traits of sexual and asexual genotypes of an aphid in environmental conditions that promote sustained parthenogenesis. According to the FTNS, and assuming that evolutionary equilibrium is not achieved (i.e., the population is always below the optimum), we predicted that the rate of increase in mean fitness in asexual genotypes should be higher than in sexual, which would be translated in heritability of fitness being significantly different than zero in asexuals, and higher than in the sexually specialized genotypes. Our results support these predictions (when using the intrinsic rate of increase as a measure of fitness), suggesting that asexual genotypes are being adapted to environmental conditions that promote parthenogenetic reproduction, relatively to the sexual genotypes. Similar results were found in rotifers (Becks and Agrawal 2011), for which a trend seems to exist for a short-term disadvantage in facultative sexual lineages relative to their asexual counterparts. In more general terms, supports for the predictions of the FTNS come from plants, where locally adapted populations exhibited higher heritabilities of fitness, compared with those from the edge of the range (Emery et al. 2011), and also from evolutionary experiments in bacteria, which showed an increase in genetic variation in fitness in time, as adaptation to local environments proceeds (Lenski and Travisano 1994).

The structure of genetic covariances showed that asexual genotypes of *R. padi* exhibited several positive genetic correlations among traits, which were absent in the sexual genotypes. This result adds to a growing record of absence of life-history trade-offs when the contrary is predicted by life-history theory (Vorburger 2005; von Burg et al. 2008; Castaneda et al. 2010c; King et al. 2011). In general, these evidences come from populations that are locally adapted or ecologically specialized, which exhibit genetic variation in resource acquisition. Hence, it appears that in addition of having comparatively high genetic variation in fitness, positive genetic correlations among life-history traits are a key feature of locally adapted populations. Interestingly, these differences in genetic (co)variances between sexual and asexual genotypes were not evident after comparing the phenotypic (co)variance matrices but after the G-matrix comparison, which indicated that one principal component was shared. This is probably due to the fact that there were no differences in signs in the genetic correlations of sexual and asexual genotypes.

As a measure of fitness, we used two common metrics for fitness determination in invertebrates: the intrinsic rate of increase (***r***) and lifetime reproduction (Huey and Berrigan 2001). Both measures are supposed to be appropriate if the population is at mutation-selection equilibrium (Roff 2010). However, when a population is in a growing phase, which is the case in our environmental conditions of sustained parthenogenesis, Roff (2010; see also Roff 2002b) has suggested that the best measure of fitness is ***r***. In our dataset, both measurements were strongly and genetically correlated only in asexual genotypes. Also, only ***r*** showed significant heritability in asexual genotypes (our interpretation as a consequence of the FTNS). It is reasonable to conclude, hence, that lifetime reproduction is perhaps not the best surrogate of fitness in experimental studies involving asexual reproducing organisms.

Few studies have evaluated SMR variation in hemipterans (Salvucci and Crafts-Brandner 2000; Castaneda et al. 2010a,b; Artacho et al. 2011), which in general have not found specific patterns associated with life-history strategies. Studies in other arthropods, however, suggest that increased reproductive output could be associated with higher energy expenditure because of the maintenance of the metabolic machinery for reproduction (Terblanche et al. 2004; Lardies and Bozinovic 2008). When analyzed as per gram basis, we did not find differences in energy expenditure between sexual and asexual genotypes. However, both energy expenditure and body mass showed positive genetic correlations with fitness traits, which suggests that asexual genotypes invest all their energy in the asexual reproduction, contrary to sexual genotypes that would be investing most of their energy in the sexual episode.

According to some authors (Lee 2002; Bacigalupe 2009), the invasion success of many species might depend more heavily on their ability to respond to natural selection than on plasticity in physiological tolerance. In other words, it would be expected that the genetic variance of life histories in invasive species is higher than in noninvasive species. This is not what the many invasive species that reproduce clonally suggests (Thompson and Eckert 2004; Voisin et al. 2005; Kim et al. 2010). Several species of aphids have lost genetic variation in populations within the invaded range (e.g., the Americas; see Castaneda et al. 2010a,b) mainly because of the reduction in the capacity for sexual reproduction. In other words, specialized genotypes for asexual reproduction are the most successfully invasive genotypes as they maximize fecundity at the expenses of survival insurance during winter. Here, the temperate climate is a key element in the invasive capacity of asexual genotypes. Our results confirm these observations, indicating that specialized asexual genotypes maximize fitness by changing their energy allocation scheme.

## Appendix

**Table A1:** Details of the 35 sexual and asexual genotypes of *Rhopalosiphum padi* used in this study, including the site, the date, and the plant of collection

				Locus							
				
Clone code	Collection site	Collection date	Plant	R10.172	R5.10	R5.29b	R5.50	R6.27	R3.171	S16b	S17b
asex1	Pleine-Fougères	12/10/2007	Wheat	195,199	256,260	172,181	304,304	178,195	225,227	147,149	159,159
asex2	Pleine-Fougères	28/09/2007	Wheat	195,199	256,271	172,181	304,328	178,195	216,225	147,149	161,165
asex3	Pleine-Fougères	28/09/2007	Wheat	195,201	258,260	178,179	312,342	188,188	218,222	147,147	159,161
asex4	Pleine-Fougères	12/10/2007	Wheat	195,201	271,260	172,181	304,336	178,195	214,225	147,149	159,161
asex5	Pleine-Fougères	28/09/2007	Wheat	201,195	254,264	174,185	304,338	178,184	207,225	147,147	159,161
asex6	Pleine-Fougères	28/09/2007	Wheat	197,201	260,260	174,178	324,340	180,184	214,225	147,147	161,161
asex7	Pleine-Fougères	28/09/2007	Wheat	195,199	258,260	174,174	328,336	190,191	216,216	147,147	159,163
asex8	Pleine-Fougères	28/09/2007	Wheat	201,201	262,271	181,200	304,310	178,180	225,247	147,147	159,171
asex9	Pleine-Fougères	12/10/2007	Wheat	195,201	271,260	174,174	304,312	178,186	216,225	147,147	159,159
asex10	Pleine-Fougères	28/09/2007	Wheat	195,197	271,271	172,181	304,366	178,195	225,225	147,149	159,161
asex11	Pleine-Fougères	12/10/2007	Wheat	195,201	271,260	172,202	304,314	186,195	225,243	147,149	159,159
asex12	Pleine-Fougères	20/11/2006	Wheat	195,201	258,256	172,185	304,304	195,195	225,225	147,149	159,161
asex13	Pleine-Fougères	20/11/2006	Wheat	195,203	256,260	172,181	304,360	178,195	225,225	147,149	165,165
asex14	Pleine-Fougères	20/11/2006	Barley	195,201	271,256	172,174	304,314	190,195	227,243	147,149	159,161
asex15	Pleine-Fougères	20/11/2006	Barley	195,199	256,271	195,181	304,328	178,195	225,225	147,149	159,159
sex1	La Chapelle Thouarault	14/04/2008	*P. padus*	195,201	262,256	172,178	304,328	195,211	227,229	147,149	159,161
sex2	La Chapelle Thouarault	14/04/2008	*P. padus*	199,201	260,271	172,204	304,336	190,195	216,225	147,147	159,161
sex3	La Chapelle Thouarault	14/04/2008	*P. padus*	195,201	271,271	164,166	304,330	178,178	216,225	147,147	159,159
sex4	La Chapelle Thouarault	14/04/2008	*P. padus*	199,201	260,271	181,181	304,310	178,180	227,247	147,147	159,159
sex5	La Chapelle Thouarault	14/04/2008	*P. padus*	199,201	254,271	181,198	304,310	178,178	225,247	147,147	159,171
sex6	La Chapelle Thouarault	14/04/2008	*P. padus*	195,201	254,256	172,185	304,304	184,195	207,225	147,149	161,165
sex7	La Chapelle Thouarault	14/04/2008	*P. padus*	199,201	266,271	174,181	304,322	178,178	225,243	147,147	165,171
sex8	La Chapelle Thouarault	14/04/2008	*P. padus*	201,201	264,271	181,185	304,310	180,184	225,247	147,147	171,171
sex9	La Chapelle Thouarault	14/04/2008	*P. padus*	199,201	262,271	174,181	328,338	178,178	207,216	147,147	161,161
sex10	La Chapelle Thouarault	30/04/2008	*P. padus*	201,201	260,262	166,172	308,347	186,195	216,243	147,147	159,161
sex11	La Chapelle Thouarault	30/04/2008	*P. padus*	201,201	260,264	174,181	304,338	178,178	207,225	147,147	159,159
sex12	La Chapelle Thouarault	30/04/2008	*P. padus*	199,201	260,260	172,200	304,342	178,195	227,227	147,147	159,161
sex13	La Chapelle Thouarault	30/04/2008	*P. padus*	199,201	271,260	172,181	304,304	180,195	207,227	147,147	159,159
sex14	La Chapelle Thouarault	30/04/2008	*P. padus*	199,195	256,258	172,208	304,334	178,195	216,225	147,149	159,161
sex15	La Chapelle Thouarault	30/04/2008	*P. padus*	201,195	258,256	172,166	304,356	195,195	216,227	147,149	159,161
sex16	La Chapelle Thouarault	05/05/2008	*P. padus*	199,201	271,271	172,181	304,310	180,195	225,247	147,147	165,171
sex17	La Chapelle Thouarault	05/05/2008	*P. padus*	199,201	258,260	164,174	304,312	178,180	207,225	147,147	159,159
sex18	La Chapelle Thouarault	05/05/2008	*P. padus*	201,201	264,271	181,174	304,338	178,180	225,225	147,147	159,161
sex19	La Chapelle Thouarault	05/05/2008	*P. padus*	195,201	262,256	181,181	304,304	178,180	225,227	147,149	159,159
sex20	La Chapelle Thouarault	05/05/2008	*P. padus*	199,201	258,271	178,181	304,350	178,193	225,235	147,147	159,163
